# Pattern of Ocular Injuries in Owo, Nigeria

**Published:** 2011-04

**Authors:** Charles Oluwole Omolase, Ericson Oluseyi Omolade, Olakunle Tolulope Ogunleye, Bukola Olateju Omolase, Chidi Oliver Ihemedu, Olumuyiwa Adekunle Adeosun

**Affiliations:** 1 Department of Ophthalmology, Federal Medical Center, Owo, Ondo State, Nigeria; 2 Department of Radiology, Federal Medical Center, Owo, Ondo State, Nigeria

**Keywords:** Eye Injuries, Pattern, Vegetative Matter, Nigeria

## Abstract

**Purpose:**

To determine the pattern of ocular injuries in patients presenting to the eye clinic and the accident and emergency department of Federal Medical Center, Owo, Ondo State, Nigeria.

**Methods:**

This prospective study was conducted between January and December 2009. Federal Medical Center, Owo is the only tertiary hospital in Ondo State, Nigeria. The eye center located at this medical center was the only eye care facility in the community at the time of this study. All patients were interviewed with the aid of an interviewer-administered questionnaire and underwent a detailed ocular examination.

**Results:**

Of 132 patients included in the study, most (84.1%) sustained blunt eye injury while (12.1%) had penetrating eye injury. A considerable proportion of patients (37.9%) presented within 24 hours of injury. Vegetative materials were the most common (42.4%) offending agent, a minority of patients (22%) was admitted and none of the patients had used eye protection at the time of injury.

**Conclusion:**

In the current series, blunt eye injury was the most common type of ocular trauma. The community should be educated and informed about the importance of preventive measures including protective eye devices during high risk activities. Patients should be encouraged to present early following ocular injury.

## INTRODUCTION

Trauma is an important cause of ocular morbidity. The pattern of ocular injuries tends to vary from one location to another and is affected by the activities of residents in a particular location.^1^ Many eye injuries are related to particular occupations and certain cultures.^2^ Some individuals by virtue of their job are prone to ocular injuries and certain activities of daily living increase the risk of eye trauma due to exposure to hazards.^3^ Despite the fact that the eyes represent only 0.27% of the total body surface area and 4% of the facial area, they are the third most common organ affected by injuries after the hands and feet.^4^ Ocular trauma is an avoidable cause of blindness and visual impairment. Ocular trauma may be caused by various objects used in different settings.^5^ Male subjects are at higher risk of ocular trauma.^6^ Even though ocular trauma has been described as a neglected issue,^7^ it was highlighted as a major cause of visual morbidity more recently.^8^

Worldwide there are approximately 1.6 million people blind from eye injuries, 2.3 million bilaterally visually impaired and 19 million with unilateral visual loss; these facts make ocular trauma the most common cause of unilateral blindness.^9^ The age distribution for serious ocular trauma is bimodal with the maximum incidence in young adults and a second peak in the elderly.^10^,^11^ Studies have shown that approximately one half of patients who present to an eye casualty department are cases of ocular trauma.^12^,^13^ Direct and indirect costs of ocular trauma are known to run into millions of dollars annually.^14^ Ocular trauma occurs frequently in developing countries and constitutes a major health problem.^15^ Even though developing countries carry the largest burden of ocular trauma, they are the least able to afford the costs.^16^,^17^ Recognition of the public health importance of ocular trauma has sparked growing interest in studies on eye injuries.^18^ The execution of this study became imperative in view of an increasing number of ocular trauma at the only eye center in the community. We designed this study to determine the pattern of ocular injuries in patients presenting to the eye clinic and the accident and emergency department of Federal Medical Center, Owo, Ondo State, Nigeria.

## METHODS

This prospective study was performed at the eye clinic and the accident and emergency department of Federal Medical Center, Owo, Ondo State, Nigeria between January and December 2009. The hospital was the only tertiary medical center in Ondo State at the time of the study. The eye center is patronized by patients from Ondo State, Nigeria and some other neighboring states.

The patients either presented directly to the hospital or were referred from other public or private hospitals. Approval was obtained from the Ethical Review Committee of the hospital prior to commencement of this study. Informed consent was obtained from each participant. All consenting patients with varying degrees of ocular injuries were enrolled in this study. The respondents were interviewed with the aid of semi-structured questionnaire by the authors. All patients underwent a comprehensive ocular examination including visual acuity (using Snellen chart), penlight, slit lamp biomicroscopy and direct ophthalmoscopy. Data were analyzed using SPSS 15.0.1 statistical software package (SPSS Inc., Chicago, IL, USA). Chi-square test was employed with statistical significance set at 0.05.

## RESULTS

One hundred and thirty-two patients including 102 male (77.3%) and 30 female (22.7%) subjects 1 to 70 years of age participated in the study. Ethnicity of the patients was Yorubas in 68.2%, Ibos in 7.6% and Hausas in 3%. The majority of the patients (82.6%) were Christian and the remaining (17.4%) were Muslim. Few patients (13.3%) had no formal education, 53 (40.2%) had primary education, 33 (25%) had secondary education, and 28 (21.2%) had tertiary education. Most patients (51.5%) were single, 47.7% were married and one subject (0.8%) was widowed. The occupation of the patients is detailed in Table 1, most of the patients (36.4%) were students.

The right eye was involved in 60 (45.5%) subjects, the left eye in 59 (44.7%) cases and the injury was bilateral in 13 (9.9%) cases. Injury occurred at home in 40 individuals (30.3%), on the road in 37 (28%), at school in 28 (21.2%), at work in 11 (8.3%), during a party in 8 (6.1%), on a farm in 7 (5.3%) and in church in 1 (0.8%). Age and sex did not affect the location at which injury was sustained (P = 0.698 and 0.072, respectively), but occupation was significantly associated with the place of injury (P < 0.001).

Most injuries (84.1%) were blunt, 6 (12.1%) were due to penetrating trauma, 2 (1.5%) eyes sustained perforating injury and 3 (2.3%) were affected by chemical burns. Age, sex and occupation did not significantly affect the type of injury (P = 0.396, 0.481 and 0.946, respectively).

The offending agents are summarized in Table 2, 42.4% were vegetative materials. Age and sex were not correlated with the type of the agent (P = 0.976 and 0.528, respectively), however occupation was significantly associated with the causal agent (P = 0.016).

A considerable proportion of patients (37.9%) presented within 24 hours, 8.3% after 48 hours, 6.8% after 72 hours, 17.4% between 3 and 7 days, and 29.5% after one week. The anatomical site of injury and level of education significantly affected the time of presentation (P = 0.007 and 0.002, respectively) but age, sex and occupation did not (P = 0.334, 0.818 and 0.287, respectively). The distance travelled by the patients to the eye center was less than 20 km in 61.4%, 20–50 km in 21.2%, 50–100 km in 12.9% and more than 100 km in 4.5%. This variable however did not significantly affect the time of presentation (P = 0.7).

Only 32 (24.2%) patients had occupational eye injury, no subject used protective eyewear at the time of injury. Only 29 (22%) cases were admitted. Most patients (51.5%) sought some form of treatment prior to presentation: 31 patients (45.6%) self administered medication, 5 (7.4%) received traditional treatment and the remaining 1 (1.5%) used a combination of treatments.

As detailed in Table 3, the most common site of injury (43.9%) was the cornea. Age and sex did not significantly affect the anatomical site of injury (P = 0.573 and 0.302, respectively) while occupation did (P = 0.027). Few patients (21.2%) had sustained injury to other parts of the face.

The majority of patients (95.5%) had good vision prior to the injury. Table 4 summarizes visual acuities of the patients at presentation and after treatment. Patient age was significantly associated with visual outcomes (P = 0.019), however sex and occupation were not (P = 0.925 and 0.169, respectively). Prognosis for restoration of vision was good in most patients (62.9%), fair in 21.2%, and poor in 15.9%.

## DISCUSSION

The predominance of Yorubas and Christians in our study is not surprising in view of their predominance in the community. There was a preponderance of male subjects in our study which is in accordance with some other reports.^3^,^19^–^21^ The predominance of ocular injuries in male subjects may be related to their aggressive and adventure seeking behavior. As reported by some other studies,^3^,^12^ most of our patients belonged to a young and active age group. This finding is understandable as such people are likely to engage in risky behaviours and activities which may predispose to ocular injuries.

The particular function of the eye has mandated an exposed location which increases the possibility of injury.^22^ The type and extent of damage sustained by the eye depends on both the mechanism and force of injury. Laterality in ocular injuries also tends to vary. There was a slight predominance of injury to the right eye (45.5%) in this study which is similar to a report by Mallika et al^21^ in Malaysia. This predominance was higher (58.5%) in a study by Okoye^3^ in Enugu, Nigeria. The reason may be the fact that most people are right handed.

Almost one third of our patients sustained injury at home. This finding is similar to the study by Mallika et al.^21^ Most of our respondents had blunt trauma which is different from the report by Okoye,^3^ stating a preponderance of penetrating trauma. Another study has reported changing patterns of eye injuries in Nigeria over the past decades during which a preponderance of war-related injuries in the early 70s was replaced by home and school related injuries as well as industrial trauma. Furthermore with the rising incidence of armed robbery and civilian-armed combats in Nigeria, gun shot injuries are becoming more common.^23^ Our study included only two cases of gun shot eye injuries. This finding may be attributed to the fact that the study population and neighboring communities enjoyed peaceful co-existence at the time of the study.

Remarkably, a significant proportion of our patients presented to the eye clinic within 24 hours of eye injury which may have contributed to the favorable outcome in most of them. Proximity to the eye center may have contributed to early presentation. Awareness of the services offered at our eye center is also likely to have prompted early presentation. The importance of increasing awareness about existing eye facilities in the populace cannot be overemphasized as this is likely to promote the utilization of such health facilities. Our findings are somehow in contrast to those reported by Babar et al^24^ in Pakistan; over 60% of their patients presented one week after eye injury. Late presentation following eye injuries has also been reported in other studies.^25^–^27^ A study by Qureshi^28^ reported that the farther the victims lived from an eye care facility, the later they presented after injury. The findings of the latter report are at variance with the current study because the distance travelled by our patients did not significantly affect the time of presentation.

It is disturbing that none of our respondents utilized protective devices at the time of injury. This finding underscores the need for educating people regarding the use of protective eyewear which may significantly decrease the magnitude of visual loss due to trauma. The impact of ocular trauma in terms of medical care, loss of income and cost of rehabilitation services clearly highlights the importance of preventive strategies.^19^

In summary there was a preponderance of eye injuries among male subjects in our study. Most patients sustained blunt eye injury and only few of them required admission. A sizeable proportion of patients presented within 24 hours after injury. Occupation and the anatomical site of injury significantly affected the time of presentation. Some respondents sustained ocular injury in their school environment. None of the patients used protective eyewear at the time of injury. The age of patients significantly affected visual outcomes. We reccomend mass education to encourage early presentation following eye injury and believe that school authorities should regulate recreational activities in school and prevent injuries to the eye and other parts of the body.

## Figures and Tables

**Table 1:** Patients’ occupation

Occupation	Frequency	Percentage
Schooling	48	36.4
Farming	24	18.2
Artisan	24	18.2
Trading	17	12.9
Civil Servant	16	12.1
Unemployed	3	2.3

Total	132	100

**Table 2:** Offending agents

	Frequency	Percentage
Metallic object	28	21.2
Finger, elbow, fist	21	15.9
Insect	11	8.3
Stone	8	6.1
Glass	3	2.3
Bullet	2	1.5
Chemical	2	1.5
Ball	1	0.8

Total	132	100

**Table 3:** Anatomical site of eye injury

	Frequency	Percentage
Lid	27	20.5
Conjunctiva	16	12.1
Cornea	58	43.9
Iris	15	11.4
Lens	6	4.5
Anterior/posterior segment	5	3.8
Cornea and lens	5	3.8

Total	132	100

**Table 4:** Visual acuity at presentation and after treatment

Visual acuity	Frequency (%)	
	At presentation	After treatment
6/5-6/18	67 (50.8)	102 (77.3)
6/18-6/60	16 (12.1)	6 (4.5)
3/60-6/60	6 (4.5)	1 (0.8)
<3/60	43 (32.6)	23 (17.4)

Total	132 (100)	132 (100)
